# Validation of the Brazilian Version of CADE-Q II to Assess Knowledge of Coronary Artery Disease Patients

**DOI:** 10.5935/abc.20180244

**Published:** 2019-01

**Authors:** Rafaella Zulianello dos Santos, Gabriela Lima Melo Ghisi, Christiani Decker Batista Bonin, Gabriela Chaves, Clarissa Machado Haase, Raquel Rodrigues Britto, Magnus Benetti

**Affiliations:** 1 Universidade do Sul de Santa Catarina (UNISUL), Florianópolis, SC - Brazil; 2 Toronto Rehabilitation Institute - University Health Network, Toronto - Canada; 3 Centro da Ciência da Saúde e do Esporte - Universidade do Estado de Santa Catarina (UDESC), Florianópolis, SC - Brazil; 4 Universidade Federal de Minas Gerais (UFMG), Belo Horizonte, MG - Brazil

**Keywords:** Cardiovascular Diseases/physiopathology, Coronary Artery Disease, Patient Education as Topic, Surveys and Questionnaires, Validation Studies, Cardiac Rehabilitation

## Abstract

**Background:**

The Coronary Artery Disease Education Questionnaire (CADE-Q), an instrument aimed at assessing patients’ knowledge about coronary artery disease (CAD), was originally developed and psychometrically validated in Brazil. It was later translated, cross-culturally adapted, and validated to English. Although both versions demonstrated good reliability and validity, new studies in the area have pointed out the need of implementing the CADE-Q with other components of cardiac rehabilitation (CR) programs, such as psychologic factors, which had not been considered in previous version and were added in the subsequent, adapted version. Thus, a second version of CADE-Q was developed in English, the CADE-Q II.

**Objective:**

to translate, culturally adapt and psychometrically validate the CADE-Q II in Brazilian Portuguese.

**Methods:**

After translation and review by a Committee of specialists in CR, a version in Brazilian Portuguese was generated and tested in 307 patients in CR. Test-retest reliability was assessed by intraclass correlation coefficient (ICC) in 49 patients; internal consistency was assessed using Cronbach’s alpha (α); and, criterion validity was assessed regarding patients’ educational level and family income. The level of significance adopted for all tests was 5%.

**Results:**

After the ICC analysis, 4 items were excluded. The questionnaire was considered internally consistent (α > 0.7). Associations were found between the mean total scores and the variables schooling (p < 0.001) and income (p < 0.001). Median total score was 53 (14) points corresponding to 65.4% of the total possible score.

**Conclusion:**

The Portuguese version of the CADE-Q II showed sufficient reliability, consistency and validity, supporting its use in future studies.

## Introduction

Cardiovascular diseases are the main cause of mortality in Brazil, as a result of both population aging and epidemiologic changes in disease,^1^^-^^3^ that contribute to high costs in health.^4^^,^^5^ Cardiac rehabilitation (CR) stands out among the recommended therapies to coronary artery disease (CAD). CR is a multidisciplinary approach for secondary prevention, that can effectively reduce rehospitalization rates by up to 18% and cardiovascular mortality by up to 26%.^6^

Most CR benefits are related to behavioral changes, and in this context, patient education is considered and important component of these programs. Education in health allows patient to understand the nature of the disease and its treatment. Consequently, inadequate understanding of the condition may lead to unwarranted emotional distress, inappropriate behavior in coping with the disease, non-adherence to treatment, and disease progression.^7^^,^^8^

Therefore, the management of chronic diseases such as CAD is crucial for secondary prevention.^9^^,^^10^ Recent studies have corroborated the benefits of educational intervention in CAD patients. That includes knowledge increase that promotes changes in self-management and health behavior resulting in improvement of quality of life,^10^^-^^12^ and potentially reduce health-related costs.^8^

Precise information on the level of cardiovascular disease knowledge in patients with this disease are essential in planning and proposing effective CR interventional programs.^7^^,^^8^ Education in health should be provided and analyzed by a simple manner, aiming at targeting many populations, including low-income patients.^13^ In this context, the coronary artery disease education questionnaire (CADE-Q) is one of the few psycometrically validated instruments available in CR, developed and validated in Brazil and also validated in other countries.^14^^,^^15^ However, the focus of CR has changed over the years,^11^^,^^16^ and a second version of the CADE (CADE-QII) was developed aiming at updating the instrument and including additional educational components, such as psychosocial health.^17^ The aim of the present study was to translate, cross-culturally adapt and psychometrically validate a Brazilian Portuguese version of the CADE-QII.

## Methods

### Study design and procedures

This study was approved by the human research ethics committee of the University Universidade Federal de Minas Gerais (approval number 1.350.973), according to the 466/12 resolution of the Brazilian National Health Council. This was an observational, cross-sectional, multicenter study, involving research centers in the cities of Belo Horizonte and Florianopolis in Brazil. Data were collected from January to May 2016.

First, the processes of translation and cross-cultural adaptation of the instrument were performed according to precise criteria previously approved by the authors, based on the protocol proposed by Guillemin et al.^18^ - (1) initial translation, (2) back translation, (3) review of the questionnaire versions built by specialist committee members, (4) pre-testing for equivalence with bilingual individuals, and (5) revision of the weighting of scores. The translated, cross-culturally adapted version of the instrument was then tested for clarity in coronary patients. The results were used to refine the CADE-QII version in Portuguese.

Second, psychometric validation was conducted. The refined instrument was administered to a larger sample of patients, participants of three CR programs in the metropolitan area of Florianopolis, Brazil (one private and two public programs) and of one public program in Belo Horizonte, Brazil. The instrument was administered by interview. The interviewers maintained a neutral position, answering questions about the study and encouraging respondents to answer all the questions. The questionnaire was readministered two weeks following the first administration in patients selected by convenience for test-retest analysis.

### Participants

For psychometric validation, we recruited participants of CR programs developed in four participating institutions, where a total of 500 patients attended every month. A convenience sample was recruited from these patients, based on availability of recruiters and patients that accepted to participate in the study. The sample was composed of 307 patients, corresponding to 61.4% of the patients attending the participating institutions. The programs lasted for at least three month and none of them had a structured educational component.

Inclusion criteria were a confirmed diagnosis of CAD or the presence of cardiovascular risk factors and signing of the consent form. Exclusion criteria were age younger than 18 years and any significant visual, cognitive or mental impairments that could limit patients’ ability to answer the questionnaire.

### CADE-QII

CADE-QII was constructed to verify the level of knowledge of coronary patients, participants of CR programs about CAD. CADE-QII evaluates the knowledge of patients for five domains consisting of medical condition, risk factors, exercise, nutrition and psychosocial risk, based on official documents and guidelines in the area.^17^ The instrument has 31 items, each item with four possible answers about one of these domains of knowledge. One of the answers is the most “correct” one, *i.e.*, the answer states complete and true information about the domain, and gets a score of 3; one of the answers is “partially correct” and gets a score of 1, and the two other options are “incorrect”, one describing incorrect information and “I don’t know”, which should be chosen when the patient is not sure about any of the previous options. Both incorrect and “I don’t’ know” options get a “0” score. The total sum is calculated, and it represents the level of patients’ knowledge about the domains.^17^ Thus, CADE-QII not only quantifies the level of knowledge about cardiovascular disease but also identify the domains where patients get the lowest scores, *i.e*., in which knowledge is deficient.

### Variables

The following characteristics of the patients participating in the psychometric validation were collected for analysis - sex, age, educational level, monthly family income, comorbidities, cardiac risk factors and previous diseases. All these characteristics were self-reported.

The Brazilian version of the CADE-QII were tested for the following psychometric properties - clarity, content validity, test-retest reliability, internal consistency and criterion validity. A descriptive analysis of total score was also performed, both per question and per domain.

### Statistical analysis

The Statistical Package for Social Sciences (SPSS), version 20.0 was used for data storage, classification and analysis. The level of significance was set at 5%. Data were excluded from analysis when more than 20% of the CADE-QII items were incomplete.

To test clarity and validity of the content, a pretest was conducted with the patients, in order to get a feedback on the items and to verify the time required to complete the questionnaire. In addition, five specialists evaluated the clarity of the CADE-QII version in Brazilian Portuguese.

The intraclass correlation coefficient (ICC) was used in the test retest reliability analysis. Values with ICC lower than 0.7 were excluded from analysis.^19^ The internal consistency of the instrument was assessed by Cronbach alpha coefficient; values above 0.70 were considered acceptable.^20^ Criterion validity was assessed by comparing CADE-QII scores with family income and educational level.^17^

Data normality was tested using the Kolmogorov-Smirnov test. Descriptive analysis of the Brazilian version of the CADE-QII was also performed; continuous variables with normal distribution were described as mean and standard deviation, and continuous variables not normally distributed were expressed as median and interquartile range. Absolute and relative frequencies were used for categorical variables. The chi-square test was used to assess associations between categorical variables.

Overall knowledge of patients was expressed as the median of the total CADE-QII score. Median scores obtained in each domain were also described.

### Translation, cultural adaptation and pretest

Initial translation of CADE-QII was made by three independent translators, aware of the objectives and underlying concepts of the study. They were asked to detect ambiguities and unexpected meanings in the Brazilian version compared with the original one. Back translation was conducted by four translators unaware of the initial objectives of the study as well as of the original version of the instrument. A commission composed of five bilingual specialists reviewed all the versions and made necessary changes according to Brazilian culture. A final version was generated, and the clarity of the questions tested in 23 coronary patients.

During translation and cultural adaptation processes, question 4 of the physical activity domain (“Three things that one can do to exercise safely outdoors in the winter are”) was adapted to the Brazilian cultural context; the change was related to the weather, the expression “hot and dry weather”, referring to summer season, were substituted for “winter” (“What one can do to exercise safely outdoors in hot and dry weather are”), which better reflects the reality of Brazil, a tropical country. No further changes were required.

Mean time required to complete the CADE-QII among participants was 22.5 ± 3.5 minutes. Mean rates for clarity of the instrument was 7.0 ± 1.77. Regarding content validity, following the administration of the instrument and discussion between patients and researchers, it was concluded that the CADE-QII clearly describes the aim of the measurements, the target population, the concepts measured and the selection of the items.

### Psychometric validation

Three hundred and seven patients that participated in CR programs completed the CADE-QII. Sociodemographic and clinical characteristics of the patients are described in Table 1. Of these patients, 228 were participants of CR in Florianopolis, and 77 in Belo Horizonte. Most patients were men (n = 200, 65.1%) and had low educational attainment (incomplete elementary school, n = 188, 61.2%). Mean age was 63.3 ± 10.4 years (minimum = 31 years old; maximum = 88 years old).

**Table 1:** Sociodemographic and clinical data, and characteristics of cardiac rehabilitation programs of patients included in the psychometric validation of the CADE-QII version in Brazilian Portuguese and association of these variables with their level of knowledge about coronary artery disease (n = 307)

Characteristics		n (%)	CADE Q II total score Median (IQR)	p*^†^*
**Sociodemographic**				
**Sex**				
	Men	200 (65.1)	54 (15.75)	0.24
	Women	107 (34.9)	51 (13)	
**Educational level**				
	Never been to school	3 (1)	59 (15)	< 0.001*
	Incomplete elementary school	88 (28.7)	45 (16)	
	Complete elementary school	39 (12.7)	48 (13)	
	Incomplete high school	19 (6.2)	51 (19)	
	Complete high school	66 (21.5)	55 (11)	
	Incomplete higher education	8 (2.6)	53.5 (11.75)	
	Complete higher education	64 (20.8)	58 (13.5)	
	Undergraduate education	20 (6.5)	60 (8.75)	
**Family income (per month)**				
	< one minimum wage	50 (16.3)	48.5 (14.25)	< 0.001*
	1 - 5 minimum wages	159 (51.8)	50 (14)	
	5-10 minimum wages	40 (13)	60 (14.75)	
	10-20 minimum wages s	32 (10.4)	56.5 (9)	
	> 20 minimum wages	26 (8.5)	57 (15.75)	
**Clinical features**				
**Comorbidities /risk factors**				
	Systemic arterial hypertension	178 (58)	52.5 (17)	0.84
	Dyslipidemias	160 (52.1)	54 (14.75)	0.11
	Obesity	77 (25.1)	51(17)	0.34
	Type I and II diabetes	76 (24.8)	54 (14.5)	0.68
	Stroke	26 (8.5)	52 (14)	0.95
	Heart failure	33 (11.0)	49 (14)	0.11
	Smoking	12 (3.9)	52 (22.5)	0.41
	Chronic obstructive pulmonary disease	11 (3.6)	55 (11)	0.12
	Peripheral arterial occlusive disease	4 (1.3)	52 (22.5)	0.96
**Acute event**				
	Myocardial infarction	222 (72.3)	52 (16.25)	0.11
**Procedures**				
	Percutaneous transluminal angioplasty	172 (56)	53.5 (16.5)	0.62
	Myocardial revascularization surgery	62 (20.2)	54 (14.75)	0.70
**Type of CR program**				
	Public	219 (71.3)	50 (17)	< 0.001*
	Private	88 (28.7)	56 (13)	

IQR interquartile range; CR: cardiac rehabilitation;

† chi-square test;

* p < 0.001

For test retest reliability analysis, 49 patients were selected by convenience and asked to complete the questionnaire again, with an interval of 15 days between the evaluations. Among these patients, 24 participated in a private CR program, and 25 in a public one. The test retest reliability was assessed by the ICC of each item, and the results are described in Table 2. The following items did not meet the minimum standards - question 4 (“A heart attack occurs”) of the medical condition domain, question 4 of factor risk domain (“The first step towards controlling a risk factor, such as blood pressure or cholesterol, is”, question 7 of nutrition domain (“How many servings of fruits and vegetables should adults consume?”) and question 5 of psychosocial risk domain (“*Chronic stress* is defined as”). These items were excluded from the Brazilian version of the CADE-QII. Thus, from the 31 items of the original version, 27 items composed the Brazilian CADE-QII in Portuguese, with a maximum score of 81 points.

**Table 2:** Median and interquartile range of CADE-QII scores by question and domain, percentage of items completed and intraclass correlation coefficient (ICC) (n = 49)

Domain	Item	Median (IQR) scores by item	Items completed (%)	ICC	Median (IQR) scores by domain
Medical condition	1. Coronary artery disease is:	3 (2)	100%	0.77	12 (6)
	2. Angina (chest pain of discomfort) occurs:	3 (2)	100%	0.82	
	3. In a person with coronary artery disease, which of the following is a usual description of angina?	3 (2)	100%	0.77	
	4. A heart attack occurs:	1 (2)	100%	0.47^‡^	
	5. The best resources available to help someone understand his/her medications are:	1 (0)	100%	0.71	
	6. Medications such as aspirin (ASA) and clopidogrel (PlavixTM) are important because:	1 (2)	100%	0.72	
	7. The "statin" medications, such as atorvastatin (LipitorTM), rosuvastatin (CrestorTM), orsimvastatin (ZocorTM), have a beneficial effect in the body by:	1 (1)	100%	0.87	
Risk factors	1. The risk factors for heart disease that can be changed are:	3 (2)	100%	0.72	10 (4)
	2. The actions that can be taken to control cholesterol levels include:	3 (0)	100%	0.82	
	3. The actions that can be taken to control blood pressure include:	3 (2)	100%	0.79	
	4. The first step towards controlling a risk factor (such as blood pressure or cholesterol) is:	0 (1)	100%	0.36^‡^	
	5. The actions to prevent developing diabetes include	1 (2)	100%	0.90	
Exercise	1. What are the important parts of an exercise prescription??	3 (2)	100%	0.71	14 (7)
	2. For a person living with heart disease, it is important to do a cardiovascular warm-up before exercising because:	1 (2)	100%	0.80	
	3. The pulse can be found:	3 (2)	100%	0.86	
	4. What one can do to exercise safely outdoors in hot and dry weather are:	3 (0)	100%	0.78	
	5. The benefits of doing resistance training (lift weights or elastic bands) include:	3 (2)	100%	0.87	
	6. If a person gets chest discomfort during a walking exercise session, he or she should:	1 (2)	100%	0.83	
	7. How does a person know if he/she is exercising at the right level?	1 (3)	100%	0.85	
Nutrition	1. What is the best source of omega 3 fats in food?	3 (0)	100%	0.85	13 (5)
	2. Trans fat are:	1 (2)	100%	0.70	
	3. What is one good way to add more fiber to your diet:	3 (2)	100%	0.80	
	4. Which of the following foods has the most salt:	3 (2)	100%	0.73	
	5. What combination of foods can help lower blood pressure?	3 (2)	100%	0.81	
	6. When reading food labels, what should one look at first?	1 (0)	100%	0.92	
	7. How many servings of fruits and vegetables should adults consume?	0 (1)	100%	0.55^‡^	
Psychosocial Risk	1. Which of the below are effective stress management techniques?	3 (0)	100%	0.92	9 (4)
	2. What stresses have been related to increased risk for heart attacks?	1 (3)	100%	0.73	
	3. Which of the following describes your best option for reducing your risk from depression:	3 (0)	100%	0.86	
	It is important to recognize "sleep apnea" because:	1 (3)	100%	0.70	
	5. "Chronic stress" is defined as:	1 (3)	100%	0.59^‡^	
Total		53 (14)	100%	0.77	-

IQR interquartile range;

‡ Items excluded from the final version in Brazilian Portuguese due to ICC values below 0.70

The internal consistency of the 27-item instrument was tested, with a Cronbach alpha coefficient of 0.78. Regarding criterion validity, as described in Table 1, patients with higher educational level (p < 0.001) and higher family income (p < 0.001) showed higher level of knowledge about the disease as compared with the other patients.

Medians and interquartile ranges of the items and domains are described in Table 2. The median total score was 53 (14) points, corresponding to 65.4% of the possible total score. The highest scores were obtained for the items: “What is the best source of omega 3 fats in food?” (“What one can do to exercise safely outdoors in hot and dry the winter are” and “Which of the following describes your best option for reducing your risk from depression”. The lowest scores were observed for the items “The first step towards controlling a risk factor (such as blood pressure or cholesterol) is”, “How many servings of fruits and vegetables should adults consume?” and “The *statin* medications have a beneficial effect in the body by”. Domains with the highest and the lowest scores were “Exercise” and “Psychosocial Risk” domains, respectively.

## Discussion

This study aimed to validate and adapt the CADE-QII in Brazilian Portuguese. During these processes, we followed strict standards, since adaptation of an instrument to be used in a country other than that in which it was developed may require more than simply semantic and idiomatic analyses.^18^ The psychometric properties - content validity, test retest reliability, internal consistency and construct validity - were established, confirming the validity of the CADE-QII for the Brazilian population.

Our results were consistent with those reported in the original validation,^17^ particularly with respect to internal consistency (Cronbach alpha of 0.91 vs. 0.78), indicating an adequate correlation between the questionnaires’ items, both in the original and in the adapted version.^17^ Nevertheless, the fact that the CADE-QII was validated in a multicentric study may have affected alpha’s value (not as high as in the original version). Another difference between the original and the adapted version was in the way the questionnaire was administered; while the adapted CADE-QII was administered by a questionnaire, in the version in English was self-administered.

With respect to criterion validity, there was a positive association of the level of knowledge about the disease with educational attainment and family income, suggesting that socioeconomic factors may be determinants to knowledge in health, which is consistent with previous studies.^14^^,^^17^^,^^20^ This is corroborated by the fact that, in the present study, there was a positive association between enrollment in a private CR program and knowledge level about the disease, reinforcing the influence of socioeconomic disparities on education in health.^21^ These data make clear the need for developing strategies aiming at overcoming the obstacles between health knowledge and patients of different social classes. In this regard, these proposals should be grounded in simple models, with high population coverage, since less educated patients are the ones who would benefit most from educational interventions.^22^

In addition, we found that patients with more comorbidities, risk factors, previous procedures and acute events did not show higher knowledge level about the disease compared with patients without these conditions. These results contrast with those found in validation of the original version, since those patients with more risk factors showed higher knowledge about the disease.^17^ These findings may be related to differences in the health education approach to the patients by the healthcare members and in patients’ ability to understand the information received in different contexts.^23^

These findings should be interpreted with caution. First, our results cannot be generalized, since our sample was selected by convenience and recruited from four CR programs only, which may limit the extrapolation of the results. Second, CADE-QII is based on education curriculum of Canadian CR programs, which are based on more rigorous educational processes than the Brazilian programs. Third, although all patients were recruited from CR programs, these were conducted at distinct centers (public and private), located in different regions of the country. Therefore, the type of educational approach and variability between the investigators may have influenced the results. Fourth, reliability analysis was performed with 49 patients enrolled in only two of the four programs. The occurrence of a response trend, hence, may not be ruled out, since data in the literature consider that a sample size of at least 50 patients is adequate for this analysis.^19^ In addition, it is possible that participants gained additional education during the 15 minute-interval between questionnaire applications, which could have also influenced the results. Fifth, CADE-QII was not developed using plain (or simple) language techniques, which may have had a negative impact on interpretation of the questions and consequently on the responses.^23^ Sixth, as previously mentioned, during validation of its original version, CADE-QII was self-administered, in contrast to the adapted version, that was administered by means of a questionnaire. Even though the interviewers have been trained, bias intrinsic to questionnaire-based methods may have influenced the answers to the instrument.

To address this, further studies are needed to validate and use the short version of the CADE-Q,^24^ as an alternative. Also, some questions were excluded during the construction of this adapted version, since they did not meet minimum standards of ICC; new validation studies proposing reformulation and inclusion of these questions are also encouraged. Also, future studies are needed to evaluate whether the Brazilian version is sensitive to longitudinal changes in assessment of patients’ knowledge before and after their participation in CR programs.

## Conclusions

This study showed that CADE-Q II version in Brazilian Portuguese version has enough reliability, consistency and validity, supporting its use in future studies to evaluate the level of knowledge of CAD patients enrolled in CR programs. This instrument could support the evaluation of the educational component in CR programs and identify knowledge domains compatible with patients’ need for information.
